# EMOTIONAL REPERCUSSIONS AND QUALITY OF LIFE IN CHILDREN AND ADOLESCENTS UNDERGOING HEMODIALYSIS OR AFTER KIDNEY TRANSPLANTATION

**DOI:** 10.1590/1984-0462/2020/38/2018221

**Published:** 2019-11-25

**Authors:** Ana Amélia Fayer Rotella, Rosemeire Aparecida do Nascimento, Maria Fernanda Carvalho de Camargo, Paulo Cesar Koch Nogueira

**Affiliations:** a Universidade Federal de São Paulo, São Paulo, SP, Brazil.; b Centro Universitário São Camilo, São Paulo, SP, Brazil.; c Hospital Samaritano, São Paulo, SP, Brazil.

**Keywords:** Renal dialysis, Kidney transplantation, Quality of life, Child, Adolescent, Psychology, Diálise renal, Transplante renal, Qualidade de vida, Criança, Adolescente, Psicologia

## Abstract

**Objective:**

To investigate the emotional repercussions and quality of life (QOL) associated with end-stage kidney disease (ESKD) in children and adolescents undergoing hemodialysis or a kidney transplant (TX).

**Methods:**

We conducted a quantitative-qualitative study. 48 children and adolescents with ESKD were interviewed; half of them underwent hemodialysis treatment, and the other half had a kidney transplantation. Their respective 48 caregivers also participated in the study. The questionnaire involved both the *Pediatric Quality of Life Inventory* and a thematic story-drawing tool. An analysis of the QOL questionnaire’s results was done by comparing the sum of points between groups and the theme-based story-drawing consisted of interpreting the data contained in the material using Freudian and Lacanian theories.

**Results:**

In the QOL questionnaires, the total score was higher in the transplanted patients and in their caregivers, suggesting a perception of better QOL after kidney transplantation. In the specific aspects of the questionnaire, physical capacity was considered superior by children who underwent transplants and their caregivers. There were no differences between the groups in the emotional, social and school aspects. However, the caregivers of the patients who had a transplant perceived a significant difference in QOL in the school aspect. In the thematic story-drawings, emotional suffering in the two analyzed groups was evidenced regardless of the treatment.

**Conclusions:**

Despite the questionnaire results suggesting that transplantation does improve some aspects of QOL, there were no differences observed between kidney replacement therapies regarding the emotional repercussion of chronic kidney disease.

## RESUMO

**Objetivo:**

Investigar as repercussões emocionais e a qualidade de vida (QV) associadas à doença renal crônica em crianças e adolescentes submetidos à hemodiálise ou ao transplante renal.

**Métodos:**

Foram entrevistadas 48 crianças e adolescentes com doença renal crônica, metade dos quais submetidos ao tratamento hemodialítico, e a outra metade, ao transplante renal. Os 48 respectivos cuidadores também participaram da pesquisa. Utilizou-se o questionário de QV *Pediatric Quality of Life Inventory* e o instrumento de desenho-estória com tema. A análise dos resultados do questionário de QV foi feita pela somatória dos pontos e a dos desenhos-estórias com tema consistiu na interpretação dos dados do material, utilizando as teorias freudiana e lacaniana.

**Resultados:**

Nos questionários de QV, a pontuação total foi superior na opinião dos pacientes transplantados e seus cuidadores, indicando percepção de melhor QV após o transplante renal. Nos aspectos específicos, a capacidade física também foi considerada superior por esse grupo. Não houve diferenças entre os grupos de pacientes nos aspectos emocional, social e escolar, porém os cuidadores dos pacientes transplantados notaram diferença na QV no aspecto escolar. Nos desenhos-estórias com tema, observou-se que o sofrimento emocional foi evidenciado nos dois grupos estudados.

**Conclusões:**

Não foram percebidas diferenças entre as terapêuticas renais substitutivas quanto às repercussões emocionais da doença renal crônica. Por outro lado, verificou-se que o transplante melhora a QV no aspecto geral.

## INTRODUCTION

Chronic kidney disease (CKD) results from an injury that leads to progressive and irreversible loss of kidney function.^1^ When the way the kidneys function is insufficient to keep a person alive, the possibilities for treatment are dialysis - hemodialysis or peritoneal dialysis - and kidney transplantation, but they do not cure the disease.^2^

The review of qualitative studies of children and adolescents undergoing hemodialysis shows feelings of insecurity around physical integrity, weakened emotional health, restricted freedom and autonomy, compromised social life and difficulties with studies.^3^ ^,^ ^4^ ^,^ ^5^

Despite the advancement of dialytic techniques, studies show that transplantation is the best pediatric therapeutic option for renal replacement, as it results in lower mortality and morbidity, better quality of life (QOL), and better growth.^6^ ^,^ ^7^

CKD during childhood and adolescence can cause psychological impairment due to the disease and its treatment. The daily life of these patients is modified by numerous restrictions, mainly physical ones, due to the specificities of the disease, which requires continuous re-adaptations in the face of the new situations, which may increase emotional and behavioral repercussions.^8^

The aim of the present study was to investigate the emotional repercussions and the consequences on the perception of QOL associated with CKD in children and adolescents in two hemodialysis and kidney transplantation units.

## METHOD

A descriptive study involving a convenience sample of 48 children and adolescents between the ages of five and 18 years old, half of whom were on hemodialysis and half of whom, had undergone a successful kidney transplant. Both groups had completed at least four months of treatment. Furthermore, their caregivers, mothers or fathers, were included, which totaled 96 individuals. Most of the participants in this study were recruited from the dialysis outpatient clinic (19 patients) and the transplant outpatient clinic (24 patients) at the *Hospital Samaritano de São Paulo*. The other five patients came from the dialysis outpatient clinic of *Hospital São Paulo*. All of the children and adolescents were enrolled in school. Participants who were undergoing psychiatric treatment, either in in-patient or who were previously diagnosed with a mental disability described in the medical record, were excluded.

The questionnaire Pediatric Quality of Life Inventory ^TM^ version 4.0 (PedsQL^TM^ 4.0) was used to address QOL issues. This questionnaire is a generic inventory validated and approved for applicability in this study. It is composed of 23 items involving physical (8 items), emotional, social and school aspects, with five items in each one. It includes self-assessment forms for children and adolescents, and parent questionnaires. The self-assessment includes the age groups of five to 7 years old (small child), eight to 12 years old (child) and 13 to 18 years old (adolescent). The parents’ questionnaire contains the same age groups and assesses their perception of their child’s QOL.

The items are similar, they differ only in terms of using appropriate vocabulary for the child’s development and the use of the first or third person. Although PedsQL^TM^ 4.0 was designed to be self-administered, in the present study, the questionnaire was applied by an interviewer to all of the participants.

The instructions ask how much each item was a problem over the past month, and respondents use a five-level scale of options: 0=never a problem; 1=almost never a problem; 2=sometimes a problem; 3=often a problem; 4=almost always a problem.^9^

Initially, children and adolescents who met the inclusion criteria answered the questionnaire according to their age, separate from their caregivers.

Then, the technique of theme-based storytelling, which was originally proposed by Aiello-Vaisberg^10^, was used in the context of emotional personality development, which uses free drawings combined with stories. When using the instrument, each person was asked to draw a person with kidney problems and to tell a story about that person. The researcher provided a white sulfite sheet, a regular pencil and colored pencils. After that, indispensable explanations were requested for the understanding and interpretation of the material from the drawing as well as in the story.

The caregivers responded to the instrument on QOL (PedsQL^TM^ 4.0), according to the chronological age of the patients and separately from their children.

The research was approved by the ethics and research committees of the two participating hospitals. The subjects’ participation in the study occurred spontaneously and the researcher offered clarifications and information to those involved. The children and adolescents signed a consent form and the caregivers signed an informed consent form.

PedsQL^TM^ 4.0 data were scored inversely and then formatted linearly into a scale of 0=100; 1=75; 2=50; 3=25; 4=0. Thus, the higher the score, the better the participant’s QOL. To analyze the results, we used frequency tables for the qualitative variables and median and interquartile range for the quantitative variables. For comparisons between groups, we used the Mann-Whitney test for quantitative variables and the Chi-square test for proportions. In all of them, the 5% limit (α <0.05) was adopted to reject the null hypothesis.

For the treatment of data from the thematic story-drawings, based on the psychoanalytic theory of Freud and Lacan, we looked for similarities in the elements present in the drawing and the story. Through the similarity of main feelings based on signifiers^11^ that emerged, expressing beliefs, values, fears, thoughts and distress, groupings could be made. Then, the themes found were interpreted. Interpretation was done through analyzing the signifiers present in the whole drawing and story.

## RESULTS

The study involved a total of 96 individuals, 48 children and adolescents and their respective caregivers. The median time and interquartile range of CKD duration of hemodialysis patients was 56 (22-108) months. And of the patients who had undergone a transplant, it was 70 (42-108) months. The main demographic and clinical data of the sample are shown in Table 1.

**Table 1:** Demographic and clinical characteristics of the studied sample (expressed as median and interquartile range).

	HD Patients **(n=24)**	TX Patients **(n=24)**	p-value	HD Caregivers (n=24)	TX Caregivers (n=24)	p-value
Age (years)	11.2 (8.1-14.2)	11.3 (8.3-15.0)	0.49	36.7 (31.6-43.6)	37.6 (34.0-43.4)	0.75
Gender (f/m)	16/8	10/14	0.08	21/3	21/3	1.00
RRT time (months)	15 (6-39)	20 (12-24)	0.67	NA	NA	
CKD time (months)	56 (22-108)	70 (42-108)	0.34	NA	NA	-

RRT: renal replacement therapy; CKD: chronic kidney disease; NA: not applicable; HD: hemodialysis; TX: transplant.

There were significant differences between the groups, the overall QOL after transplant was higher than that of patients undergoing dialysis, a condition perceived by both patients and caregivers. By specifying each item under physical capacity, patients who had undergone transplants showed better physical conditions. It is also noted that according to the responses of caregivers of patients with transplants, after the transplant, school activity was identified to be better than that of patients undergoing dialysis. Regarding the emotional and social aspects, it was observed that there were no significant differences between the groups, both regarding patients and their caregivers. Tables 2 and 3 represent the results of the QOL questionnaire.

**Table 2:** Results and descriptive level of comparisons between patient groups, according to the Mann-Whitney test (median and interquartile range).

	HD Patients (n=24)	TX Patients (n=24)	p-value
Total score	60.3 (52.2-67.4)	69.5 (59.2-79.3)	0.042
Physical capacity	70.3 (56.3-81.3)	81.3 (70.3-93.8)	0.011
Emotional aspect	60.0 (50.0-72.5)	60.0 (47.5-75.0)	0.909
Social aspect	62.5 (45.0-75.0)	70.0 (50.0-77.5)	0.394
School activity	55.0 (37.5-70.0)	70.0 (50.0-80.0)	0.103

HD: hemodialysis; TX: transplant.

**Table 3:** Results and descriptive level of comparisons between groups of caregivers, according to the Mann-Whitney test (median and interquartile range).

	HD Caregivers (n=24)	TX Caregivers (n=24)	p-value
Total score	64.7 (50.0-73.9)	77.7 (70.1-82.6)	0.005
Physical capacity	65.6 (46.9-87.5)	92.2 (79.7-93.6)	0.004
Emotional aspect	60.0 (42.5-77.5)	65.0 (55.0-70.0)	0.413
Social aspect	70.0 (52.5-90.0)	80.0 (67.5-85.0)	0.236
School activity	62.5 (40.0-70.0)	70.0 (60.0-80.0)	0.029

HD: hemodialysis; TX: transplant.

From the participants’ drawing-stories, eight sentences (themes), which represent the observed signifiers were formulated, namely:

- Hemodialysis group - what has the disease done to me?; the kidney is my scar; transplant my salvation; and I have nothing.
- Transplant group - what has no remedy will never be cured; even with problems, keep going; there is another life; and only God heals.

Most of the patients made self-referential drawings, demonstrating that the research participant’s feelings matched their creation in the themed story-drawing. There was no need to separate the results. Although the adolescents were able to more precisely say that they were suffering, the two groups presented significant similarities regarding the representation of their suffering. The disease overlapped with age, and suffering put all of them in a similar condition.

### Hemodialysis group

**Figure 1:**
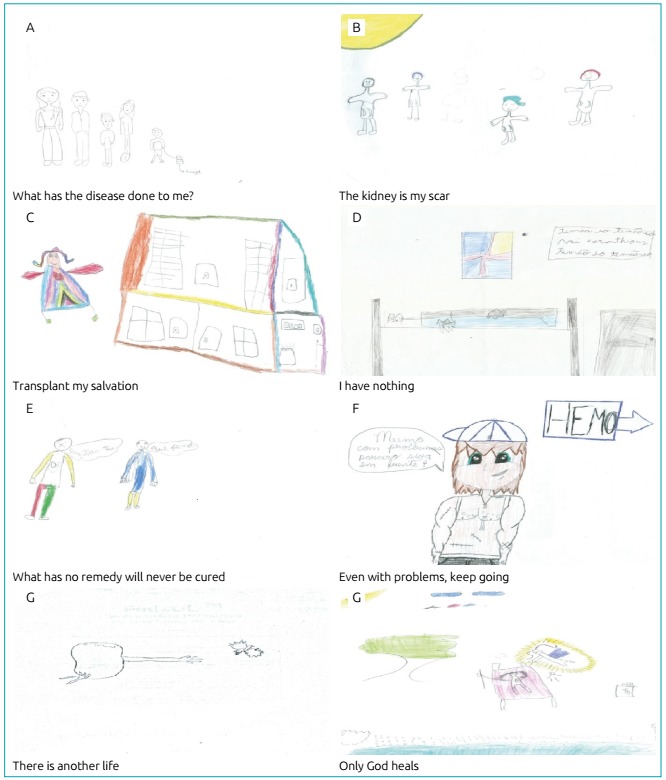
Themes that represent the signifiers observed in the thematic story drawings.

The theme “What has the disease done to me?” was made up of ten patients with similar signifiers: sadness, loneliness, and tiredness related to the kidney disease. They realize that instead of playing, having fun and walking, they spend most of their time restricted to a machine: “*They are sad and tired chronic kidney patients. There’s also the angry ones, those who do not take medicine and then have consequences, such as a crooked leg (...) We realize that others who do not have to do hemodialysis can eat, go to other places, while we can’t*”*.* Figure 1A illustrates this theme.

The theme “the kidney is my scar” totaled five patients with signifiers of kidney, dialysis and disease. There is a belief that their lives are made to live with the consequences of kidney disease. Some consider the fact that they were born in families with health problems: “*The teenager cannot rap his head around the disease. The teenager thinks about why he has this disease. [...] in the drawing are two children and two teenagers, all with marked kidneys*”, which can be represented by Figure 1B.

The theme “transplant my salvation” encompassed five patients and highlighted the signifiers of transplants, feeling well and returning to life before the kidney disease. It is based around the belief that people that have transplants will have a life without restrictions: “*I met S. at the hospital doing kidney treatment [...], now she feels good about having a transplant*”*.* This theme is exemplified by Figure 1C.

The last theme of the group, “I have nothing”, contemplated four patients and it revolved around the signifiers of: being a normal person and eating without restrictions. Here denial is a defense, it is preferable not to do anything that can cause distress: “*He has a normal life [...] played ball with his friend M., then they went to the pool, read books, ate ice cream, ate snacks, played some more and went to sleep*”*.* This can be represented by Figure 1D.

### Transplant group

The theme “what has no remedy will never be cured” corresponded to eight patients with significant impotence and loneliness. They appeared to feel weak even after the transplant: “*He does not have a kidney [...], he couldn’t play because he didn’t have a kidney*”, which can be shown in Figure 1E.

The “even with problems, keep going” theme came up with eight patients who point out signifiers of feeling happy and moving on: “*I don’t know who this boy is, but I decided to draw him. He is full of problems, full of scars, cares for the body even without its beauty, because the body has many imperfections*”*.* In Figure 1F, there is an example of a patient who fits this theme.

The theme “there is another life” was composed of six patients who affirmed signifiers of freedom, with the perception that, after having a transplant, life is better: “*I drew a kidney releasing a dove of freedom*”*.* In Figure 1G, one can get a more accurate idea of this signifier.

The theme “only God heals” included two patients and the signifiers are God and miracle. It established the belief that medicine and treatments do not cure, well-being depends on divine will, thus a kidney patient must have faith: “*I drew a girl, she was transplanted with health and energy. God gave her a kidney, a miracle. A* Figure 1H *illustrates this theme well.*

## DISCUSSION

The results of the questionnaire pointed out the differences in the perception of QOL between hemodialysis patients and patients who received transplants, as well as their caregivers’ perception of the patients’ QOL. In the themed story-drawings, emotional distress was observed in both groups.

In the QOL questionnaire, the total score, which reflects the perception of overall QOL, was higher in both patients who had received transplants and their caregivers, suggesting a perception of better QOL after kidney transplantation. The sub-items of this instrument indicated that this effect is more important when considering physical capacity, which was considered better than that of individuals on hemodialysis, both in terms of self-perception and caregiver perception. With regard to school, only the caregivers of transplant patients noticed improvement in their children’s QOL. Emotional and social aspects did not differ, suggesting that the benefit of transplantation in these areas may be more modest than what was observed in regard to physical capacity.

Patients’ social impairments can be explained by the demands imposed by the treatment and difficulties inherent to the disease itself. It was observed that family and close friends often form the patients’ only social networks.^12^ Some patients suffer when having to reveal that they have chronic renal disease, and they feel ashamed of the arteriovenous fistulas in their arm or their dialysis catheter.^13^ In addition, children and adolescents with CKD may present limitations with regard to height, delay in the development of sexual characteristics, bone deformities, and scars, which arouse the curiosity of others. They may be called out as sick people, which can lead to embarrassing situations and aggravate isolation, even for transplant recipients.^13^

School performance was perceived to be more affected only by caregivers of individuals on hemodialysis, which may reflect differences in patients and caregivers’ school expectations. Children and adolescents with chronic conditions, as well as their families, often occupy their time and thoughts on treatment, neglecting other aspects of their lives. This often causes school dropout, frequent absences, reduced interest and lack of motivation with learning. There is a need for schools to know and participate in what happens to its students, so that they can provide comfort and act as another support network.^14^

Due to social difficulties and problems with the health system in our country, transplants are performed later in the pediatric population, which may be one of the reasons for the lack of differences in QOL between the two groups of patients regarding emotional, social and school aspects. Findings from other studies, in relation to the treatments used, are similar to the results of this study: the worst QOL scores were found in dialysis patients, and adolescents on dialysis reported worse QOL, with more physical impairment compared to patients with a working graft transplant.^15^ ^,^ ^16^ The children going through puberty complained of feeling different and not accepted by their peers. They also reported an intense desire to relate.^17^ ^,^ ^18^ ^,^ ^19^ This may be associated with the scores found in the two groups studied here, with regard to social and emotional impairment. There is also a study that showed no differences in any of the QOL scores, regardless of the treatment between the groups of patients with CKD. Overall QOL was compromised compared to a control group. In contrast, a recent survey of Spanish and Portuguese children and adolescents reported different results regarding the positive perception of QOL in hemodialysis treatment and kidney transplantation. Even from the analysis of the material said by the research subjects, positive feelings emerged in most of the patients studied.^21^

Among the limitations of this study are the instruments used and the lack of a control group with healthy children and adolescents to serve as a parameter. The questionnaire provides a generic estimation of patients’ QOL because, when it was submitted to the ethics councils for research approval, there were no standardized and updated instruments for the specificity of CKD in the area of pediatric QOL that had been properly translated, adapted and tested in the Brazilian context. After the start of this research, the CKD-specific PedsQL instrument was validated for Portuguese. This could have provided us with greater depth in the results found. Another restriction refers to the fact that the research was conducted in only two dialysis and transplant centers. Multicenter studies are needed in order to prospectively monitor the phases of CKD treatment.

Nevertheless, it is impossible to reduce the complexity of the concept of QOL into a questionnaire, because the concept is interposed with health, which means satisfaction and well-being in the physical, psychic, socioeconomic and cultural spheres.^23^ It is also important to value the material found in the themed story-drawings, which present the emotional repercussions experienced by the patients. Thus, we emphasize the fact that we have a quantitative and qualitative approach, which gives us a greater scope of the theme studied. In the theme “What has the disease done to me?” there was a feeling of exclusion, which may be due to the fact that patients have peculiar characteristics because of the disease. This most often makes them feel different and called out as being sick. Often a patient’s height does not match their chronological age, their skin is pale and they have a vascular, fistula or catheter access, which can cause embarrassment.^14^ In the subsequent theme, “the kidney is my scar”, we observed children and adolescents that identify with the idea of living as if the disease were the only thing in their life. The repercussions that come from such a representation may be felt on a psychic and organic level. In the theme “transplant my salvation”, the patients portrayed their expectations regarding the transplant, because they envisioned being able to resume their lives without having to undergo hemodialysis any longer. The best-known treatment for CKD is kidney transplantation, as it is the most physiological and painless method, and also because it offers greater freedom. Survival is longer than in dialysis patients, which is why they expect better QOL with transplantation.^24^

In this study, it was observed that, in transplant patients, some aspects of QOL such as emotional, social and school related aspects were similar to those undergoing hemodialysis. In the story-drawing themes, it is possible to see the suffering that comes from treatments for CKD in both groups. However, in the transplant recipients, it was confirmed that there are indeed beneficial changes, as observed in the following reports: “Anyone can see the difference. Today I have more freedom than I had before [...]”, “[...] now, after the transplant, everything is fine. I learned to ride a bicycle without training wheels [...]”,“ [...] you feel good, you can travel, you can drink plenty of water, you can eat everything [...] ”. However, such benefits do not mean that the health problems of these patients are solved, as noted in the following reports: “After the transplant, I’m still alone,” “I drew what I looked like before I had the kidney problem and I was happy. Afterward, I started throwing up and feeling bad.”

In the last item of those who undergo hemodialysis, “I have nothing”, it was found that the disease can cause patients to use defense mechanisms in order to better live with the anguish, displeasure and anxiety arising from the illness. The defense mechanism of denial is understood as the way in which an individual comes into contact with his or her previously hidden desires and feelings, but unconsciously defends himself or herself against these feelings by denying that he or she has them.^25^

In addressing the transplant group on the theme “what has no remedy will never be cured”, most highlighted having suffered from the kidney disease, although some emphasized the fact that life is better now. It is worth noting the patients’ fragility in the face of changes imposed by CKD within the theme “even with problems, keep going.” In the end, the disease does not prevent the patient from wanting to move on.^26^

In “there is another life,” the patients attempt to give a new meaning to the scars of their history. They have a degree of satisfaction after having had difficult times related to the disease, leading them to consider their current experience as a new life. Transplantation is often experienced as a return to control over one’s life.

In the theme “only God heals,” one can think of religion from the perspective of alleviating the pain of human helplessness. Freud emphasized the importance of substitute satisfaction, that is, imagination, experiences with knowledge, artistic satisfaction, and religious beliefs, which are reaffirmed by the process of sublimation.^27^

In conclusion, the QOL instrument demonstrated the difficult issues that patients need to work on, which are emotional, social and school aspects. At the same time, thematic story-drawings allowed us to recognize the feelings of loneliness, sadness, tiredness, helplessness and freedom. Apprehension regarding the experiences lived and reported by the patients of this study provokes reflections about the individualization of care, given the understanding that people can interact differently when affected by CKD or when undergoing kidney transplantation. Kidney transplantation can help patients from a clinical and psychological point of view, but not necessarily. And they should not be considered the only solution, because even after transplants, children and adolescents are still overcome by the subjectivity of their memories, pain, hospitalizations, consultations and also because they continue to take medicine and see doctors.
